# Medical malpractice in hospitals—how healthcare staff feel

**DOI:** 10.3389/fpubh.2023.1080525

**Published:** 2023-06-01

**Authors:** Shang-Feng Tsai, Chieh-Liang Wu, Yu-Ying Ho, Pei-Yi Lin, Ai-Chu Yao, Ya-Hui Yah, Chia-Min Hsiao, Yu Huei You, Te-Feng Yeh, Cheng-Hsu Chen

**Affiliations:** ^1^School of Medicine, National Yang-Ming University, Taipei, Taiwan; ^2^Division of Nephrology, Department of Internal Medicine, Taichung Veterans General Hospital, Taichung, Taiwan; ^3^Department of Life Science, Tunghai University, Taichung, Taiwan; ^4^Department of Post-Baccalaureate Medicine, College of Medicine, National Chung Hsing University, Taichung, Taiwan; ^5^Department of Critical Care Medicine, Taichung Veterans General Hospital, Taichung, Taiwan; ^6^Department of Nursing, Taichung Veterans General Hospital, Taichung, Taiwan; ^7^Office of Social Work, Taichung Veterans General Hospital, Taichung, Taiwan; ^8^Department of Healthcare Administration, Central Taiwan University of Science and Technology, Taichung, Taiwan

**Keywords:** medical malpractice, medical dispute, medical malpractice stress syndrome, posttraumatic stress disorder, Stanford Acute Stress Reaction Questionnaire, Impact of Event Scale-Revised

## Abstract

**Introduction:**

Literature is limited on quantified acute stress reaction, the impact of event scale on medical staff when facing medical malpractice (MMP), and how to individually care for staff.

**Methods:**

We analyzed data in the Taichung Veterans General Hospital from October 2015 to December 2017, using the Stanford Acute Stress Reaction Questionnaire (SASRQ), the Impact of Event Scale-Revised (IES-R), and the medical malpractice stress syndrome (MMSS).

**Results and Discussion:**

Of all 98 participants, most (78.8%) were women. Most MMPs (74.5%) did not involve injury to patients, and most staff (85.7%) indicated receiving help from the hospital. The internal-consistency evaluations of the three questionnaires showed good validity and reliability. The highest score of IES-R was the construct of intrusion (30.1); the most severe construct of SASRQ was “Marked symptoms of anxiety or increased arousal,” and the most were having mental and mild physical symptoms for MMES. A higher total IES-R was associated with younger age (<40 y/o), and more severe injury on patients (mortality). Those who indicated receiving very much help from the hospital were those having significantly lower SASRQ sores. Our study highlighted that hospital authorities should regularly follow up on staff’s response to MMP. With timely interventions, vicious cycles of bad feelings can be avoided, especially in young, non-doctor, and non-administrative staff.

## Introduction

Medical malpractice refers to a negligent act, omission, or unintentional harm, injury, or death to a patient caused by a medical or healthcare professional. Unfortunately, incidents of medical malpractice are on the rise worldwide (1, 2). In America, 7.4% of physicians receive a malpractice claim every year, with 1.6% having to pay for the claim (3). According to an insurance company in Germany, 4,500 out of 108,000 (4.2%) insured doctors are involved in medical disputes each year, with 30% ending in settlement and 10% appearing in a civil court (4). In England, medical malpractice cases have also increased more than double over a 5-year period (from 2007 to 2012) (2). In Taiwan, such incidence has also increased steadily (5, 6), and the number of civil cases is nearly four times higher in 2007 than in 2004. The negative side effects impact the healthcare system, with inadequate physician subspecialties due to more and more judicial processes (7). Physicians have dramatically increased their use of defensive medications over the past 20 years in order to mitigate the risk of malpractice lawsuits (8). However, the use of such medications can lead to overdiagnosis and overtreatment, which can be considered a form of medical error. The defensive practice also results in increased direct and indirect medical costs (9–11). The combination of medical errors, malpractice, and defensive medicine is often referred to as the ill-fated triad (8). Most recent studies on medical malpractice focus on the history of physicians’ malpractice claims (12), the workload of medical staff, communication style (13, 14), specialties cattery (5, 15), levels of the hospital, and the court making the final judgment (3, 5, 16). There is limited research on the emotional experiences of medical staff who have been involved in cases of medical malpractice. Furthermore, like in Japan (17), medical malpractice-related mortality or injury is a crime under Taiwan’s Criminal law code. The United States, Canada, and the United Kingdom all deal with medical practice as a common law heritage. The United States, Canada, and the United Kingdom handle medical malpractice cases under the common law legal system, while Taiwan treats it as a criminal matter. This approach can create significant pressure on medical staff during the practice of medicine. Despite this, there is a lack of research in Taiwan exploring the emotional experiences of medical staff who have been involved in cases of medical malpractice.

Medical malpractice stress syndrome (MMSS) is a term coined only recently to describe what happens when medical staff face medical malpractice (18). Apart from anxiety and depression, MMSS includes perturbations in inflammatory states, immune dysregulation, and endocrine dysfunction (19). The IES-R questionnaire was first created by Weiss et al., in 1997 to evaluate the impact of a specific event (20). IES-R is a good tool for evaluating individuals with or without posttraumatic stress disorder (PTSD) (21). The Stanford Acute Stress Reaction Questionnaire (SASRQ) was created >20 years ago (22) and has been a valid and reliable measure of acute stress (22). Undoubtedly, medical staff who are involved in medical malpractice are experiencing PTSD and acute stress. However, no study using SASRQ and IES-R to evaluate their feelings or response to medical malpractice has been reported.

Medical malpractice is a global issue, and in Taiwan, medical staff who are involved in medical malpractice may face criminal charges under the Criminal Law Code. Despite the gravity of the situation, no studies have been conducted on the emotional experiences of healthcare staff facing medical malpractice charges. Due to the lack of a definitive list to describe medical malpractice stress, we employed the MMES, SASRQ, and IES-R to investigate the psychological effects of medical malpractice on healthcare staff. We analyzed potential background factors associated with anxiety or stress, as well as the extent to which hospital authorities provided support. By gathering this information, we hope to make recommendations to hospital authorities on effective measures to support medical staff and deliver timely interventions.

## Materials and methods

### Institute and participants

This is a retrospective study aimed at evaluating the impact of medical malpractice on the staff at the Taichung Veterans General Hospital (TCVGH). TCVGH is a public medical center in Taichung City, Taiwan, with 1,500 beds and approximately 5,500 employees. It provides safe and high-quality medical services and serves as the referral hospital for critically ill and complex cases. TCVGH is ranked number one in Taiwan based on the case-mix index, reflecting the complexity and risk level of diseases and treatments. The hospital provides patient-centered care and has multiple cross-department centers for integrated care. Given this background, the impact of medical malpractice and legal proceedings on staff is a major concern for the hospital authorities, and monitoring, early intervention, and out-of-court settlements are important issues. In a previous study (23), clinical risk management could be successfully applied to this topic. Besides, the reporting system is a vital tool for clinical risk management according to Italian experiences (24, 25).

In October 2015, TCVGH initiated an early warning and reporting system to address potential medical disputes and litigations. The primary objective of this system was to monitor and provide early intervention to minimize medico-legal issues and care for employees. For this study, data was collected on all staff, including doctors and non-doctors, who had experienced medical malpractice issues from October 2015 to December 2017. Participants were asked to complete three questionnaires, namely, the Stanford Acute Stress Reaction Questionnaire (SASRQ), the Impact of Event Scale-Revised (IES-R), and the Medical Malpractice Stress Syndrome (MMSS) questionnaire to evaluate the impact of medical malpractice. Only those who completed all three questionnaires were included in the analysis. The study was approved by the Ethics Committee of the Taichung Veterans General Hospital (IRB number: CE18097A), and all methods were performed in accordance with relevant guidelines and regulations.

### Definition of variables and outcomes (IES-R, SASRQ, and MMSS)

The baseline data that we collected from participants included their age, gender, degree (bachelor’s, master’s, or Doctor of Philosophy), religion, and marital status (unmarried, divorced, widowed, or married). We also gathered information on their work status and medical malpractice, including whether they were in an executive role or not, their job type (doctor or non-doctor), job tenure (less than 2 years, 2–10 years, 10–20 years, or more than 20 years), the severity of injury to patients, and whether they received any help from the institute (not at all, a little, or a lot). The definition of “help” is based on staff members’ subjective feelings. At our institute, when medical staff members are involved in medical malpractice, the chief of their department and social workers reach out to provide counseling and mental support. If there are concerns about a lawsuit, lawyers from our institute also provide further support.

### Questionnaire on the Impact of Event Scale-Revised (IES-R)

IES-R was created by Weiss et al. in 1997 to evaluate the impact of a specific event (20). The major characteristic of PTSD is the distressing oscillation between intrusion and avoidance. There are three constructs (philosophy) (a total of 22 questions) of evaluation, including intrusion, avoidance, and hyperarousal. This IES-R has been cited 6,697 times and is widely used to evaluate PTSD. The score of each question is zero for the least severe and four for the most severe conditions. The total score ranges from zero to 88. If the total score is >32, the diagnosis of PTSD is strongly suggested (26). In our current study, we used the Chinese version of the Impact of Event Scale-Revised (CIES-R), which has a high Cronbach’s alpha score (0.83–0.89). The high Cronbach’s alpha score of CIES-R indicates an acceptable high internal-consistency reliability in our population.

SASRQ was created >20 years ago and it is the most widely used questionnaire of this kind worldwide (22). It has different versions and has been used for >90 publications (27). It was initially a 98-item questionnaire, and subsequently reduced to a 30-item questionnaire to fit DSM-IV Acute Stress Disorder criteria. The new version has high predictive power for PTSD. It includes four groups: dissociation, re-experiencing of the trauma, avoidance of reminders of the trauma, and marked symptoms of anxiety or increased arousal. It is also used to evaluate impairment in social or occupational functioning. For each item of the questionnaire, the least severe score is zero, and the most severe score is five (5-point Likert scale). SASRQ represents a good research checklist with very good internal consistency (0.80–0.95) and test–retest reliability (0.69) (22). A total score (range 0–150) is calculated, and high scores suggest serious acute stress. In this study, we used the Chinese version of SASRQ, which was also used in our previous study (28).

### Questionnaire of medical malpractice stress syndrome (MMSS)

MMSS is considered to be one aspect of PTSD, and healthcare staff may experience it in the form of psychological, physical, and behavioral trauma. As there is no single definitive list of symptoms, we used the most frequently cited indications for our study (26, 29). They included mental symptoms (14 questions), physical symptoms (15 questions), and the impairment of interpersonal relationships (5 questions). All questions had to be responded to with only “yes” or “no.”

### Statistical analyses

The presentation of continuous variables was done as mean ± standard deviation, while the presentation of categorized variables was done as a percentage (%). To compare differences between continuous variables, we used the independent samples student *t*-test. To evaluate possible associated factors of outcomes for medical malpractice, we used Analysis of Variance (ANOVA). To evaluate the internal consistency reliability of IES-R and SASRQ, we used Cronbach’s Alpha score, while for MMSS, we used Kuder–Richardson 20. All data were analyzed using IBM SPSS Statistics version 23, and statistical significance was set at *p* < 0.05.

## Results

### Baseline characteristics of all participants

The data from the remaining 98 participants were analyzed after two participants did not complete all questionnaires. Most participants (78.8%) were women, and distributions across age ranges were similar: 39.8% of <29 y/o, 24.5% of 30–39 y/o, and 35.7% of ≥40 y/o (Table 1). Most of them were bachelor’s degree holders (77.6%), non-executive workers (86.9%), and non-doctors (79.6%). More than one-third of them had a job tenure between 2 and 10 years. Most medical malpractices (74.5%) occurred without patient injury. Most staff indicated receiving help from the hospital (21.4% with a lot of help, and 64.3% with a little help).

**Table 1:** Baseline data of participants.

Variable	Number	Percentage
Gender		
Female	77	78.6
Male	21	21.4
Age (y/o)		
<29	39	39.8
30–39	24	24.5
≥40	35	35.7
Degree		
Bachelor	76	77.6
Master, or Doctor of Philosophy	22	22.4
Marriage		
Unmarried, divorced, widower or widow, or	53	54.1
Married	45	45.9
Executive class		
No	85	86.7
Yes	13	13.3
Job		
Doctor	20	20.4
Nurse	78	79.6
Job tenure (years)		
<2	24	24.5
2–10	34	34.7
10–20	17	17.3
>20	23	23.5
Religion		
No	49	50.0
Yes	49	50.0
Any injury of this medical malpractice to patients		
No injury	73	74.5
Major injury	11	11.2
Mortality	14	14.3
Did the help provided by the hospital work for you?		
Not at all	14	14.3
A little	63	64.3
Very much	21	21.4

Internal consistency reliability evaluations of the three questionnaires (IES-R, SASRQ, and MMSS) showed good validity and reliability (Table 2). All Cronbach’s Alpha scores in IES-R were > 0.9, specifically 0.969 for all IES-R, 0.936 for the construct of intrusion, 0.906 for the construct of avoidance, and 0.912 for the construct of hyperarousal. All Cronbach’s Alpha scores in SASRQ also showed good validity: 0.972 for all SASRQ scores, 0.920 for the construct of dissociation, 0.844 for the construct of re-experiencing the trauma, and 0.929 for the construct of avoidance of reminders of the trauma. Similarly, Kuder–Richardson’s 20 scores for MMS showed good validity: 0.914 for mental symptoms, 0.912 for physical symptoms, and 0.789 for interpersonal relationships.

**Table 2:** Scores of different constructs (philosophy) from SASRQ, IES-R, and MMSS.

Variables	Range	Min	Max	Mean	Standard deviation	Standardized mean	Cronbach’s Alpha
IES-R	0 ~ 88	0	88	25.3	17.5	28.8	0.969
Intrusion	0 ~ 32	0	32	9.6	6.6	30.1	0.936
Avoidance	0 ~ 32	0	32	9.1	6.2	28.4	0.906
Hyperarousal	0 ~ 24	0	24	6.6	5.4	27.5	0.912
SASRQ	0 ~ 150	0	118	61.4	29.4	52.0	0.972
Dissociation	0 ~ 50	0	40	18.0	9.6	45.1	0.920
Re-experiencing the trauma	0 ~ 30	0	27	13.5	6.2	50.2	0.844
Avoidance of reminders of the trauma	0 ~ 30	0	28	12.3	7.1	43.8	0.929
Marked symptoms of anxiety or increased arousal	0 ~ 30	0	22	13.8	6.6	62.9	0.908
Impairment in social or occupational functioning	0 ~ 10	0	10	3.7	2.2	37.0	0.788
MMSS							KR 20
Mental symptoms							0.914
Physical symptoms							0.912
Interpersonal relationship							0.789

Cronbach’s Alpha or Kuder–Richardson 20: for the evaluation of internal consistency reliability.

### Responses of medical staff to medical malpractice

Detailed results of the IES-R for PTSD are shown in Table 3, with the summary listed in Table 2. The standardized mean value of all IES-R scores was 28.8. Among them, the highest score was the construct of intrusion (30.1), followed by the construct of avoidance (28.4) and hyperarousal (27.5). In general, detailed impact scores of medical malpractices were not high (Table 3). Of all the 22 events’ scores, most were less than moderate severity at <1.5 of the mean score, except two items: “Item 1: Any reminder brought back feelings about it (1.58 of the score)” and “Item 21: I felt watchful or on-guard (1.73 of the score).”

**Table 3:** Results of the Impact of Event Scale-Revised (IES-R) for post-traumatic stress disorder (PTSD).

Construct	Questions	Mean	Standard deviation
	Total score	25.32	17.47
I	1. Any reminder brought back feelings about it.	1.58	1.04
I	2. I had trouble staying asleep.	1.19	1.03
I	3. Other things kept making me think about it.	1.44	1.07
H	4. I felt irritable and angry.	1.23	1.08
A	5. I avoided letting myself get upset when I thought about it or was reminded of it.	1.42	1.03
I	6. I thought about it when I did not mean to.	1.37	0.96
A	7. I felt as if it had not happened or wasn’t real.	0.89	0.96
A	8. I stayed away from reminders about it.	1.23	0.95
I	9. Pictures about it popped into my mind.	1.26	0.94
H	10. I was jumpy and easily startled.	0.95	1.08
A	11. I tried not to think about it.	1.27	1.05
A	12. I was aware that I still had a lot of feelings about it, but I did not deal with them.	1.14	0.91
A	13. My feelings about it were kind of numb.	0.96	0.99
I	14. I found myself acting or feeling as though I was back at that time.	0.85	0.93
H	15. I had trouble falling asleep.	1.02	1.11
I	16. I had waves of strong feelings about it.	1.10	1.00
A	17. I tried to remove it from my memory.	1.16	1.03
H	18. I had trouble concentrating.	0.85	0.97
H	19. Reminders of it caused me to have physical reactions, such as sweating, trouble breathing, nausea, or a pounding heart.	0.82	1.05
I	20. I had dreams about it.	0.84	0.97
H	21. I felt watchful or on guard.	1.73	1.17
A	22. I tried not to talk about it.	1.02	1.07

A, avoidance; H, hyperarousal; I, intrusion.

Detailed results of the impact of SASRQ on medical malpractice are shown in Table 4 and the summary is listed in Table 2. The standardized mean value of all SASRQ was 52.0, which represented the level of moderate severity (Table 2). The most severe construct of SASRQ was “Marked symptoms of anxiety or increased arousal” (62.9 of the standardized mean value). The least severe construct of SASRQ was “Impairment in social or occupational functioning” (37.0 of the standardized mean value). Of all the 30 items of SASRQ (Table 4), the highest score was Item 23: I would suddenly act or feel as if the medical malpractice is happening again” (3.17 of the mean score), followed by “Item 2: I felt restless” (2.69 of the mean score) and “Item 1: I had difficulty falling or staying asleep” (2.56 of the mean score). The lowest score was “Item 10: I did not have the usual sense of whom I am.” (1.16 of the mean score), followed by “Item 13: I experienced myself as though I were a stranger.” (1.45 of the mean score), and “Item 16: I had problems remembering important details about the medical malpractice.” (1.54 of the mean score).

**Table 4:** Results of the Stanford Acute Stress Reaction Questionnaire (SASRQ).

Construct		Mean	Standard deviation
	Total scores	61.36	29.43
M	1. I had difficulty falling or staying asleep	2.56	1.27
M	2. I felt restless	2.69	1.26
D	3. I felt a sense of timelessness.	2.23	1.32
D	4. I was slow to respond.	2.34	1.18
A	5. I tried to avoid feelings about the medical malpractice.	2.29	1.29
R	6. I had repeated distressing dreams of the medical malpractice.	1.76	1.25
R	7. I felt extremely upset if exposed to events that reminded me of an aspect of the medical malpractice.	2.55	1.41
M	8. I would jump in surprise at the least thing.	1.94	1.40
	9. The medical malpractice made it difficult for me to perform work or other things I needed to do.	2.02	1.27
D	10. I did not have the usual sense of who I am.	1.16	1.19
A	11. I tried to avoid activities that reminded me of the medical malpractice.	2.04	1.40
M	12. I felt hypervigilant or “on edge”	2.40	1.41
D	13. I experienced myself as though I were a stranger.	1.45	1.29
A	14. I tried to avoid conversations about the medical malpractice.	1.95	1.38
R	15. I had a bodily reaction when exposed to reminders of the medical malpractice.	1.81	1.40
D	16. I had problems remembering important details about the medical malpractice.	1.54	1.29
A	17. I tried to avoid thoughts about the medical malpractice.	1.94	1.42
D	18. Things I saw looked different to me from how I know they really looked.	2.12	1.30
R	19. I had repeated and unwanted memories of the medical malpractice.	1.93	1.43
D	20. I felt distant from my own emotions.	1.79	1.28
M	21. I felt irritable or had outbursts of anger.	2.31	1.38
A	22. I avoided contact with people who reminded me of the medical malpractice.	2.00	1.38
R	23. I would suddenly act or feel as if the medical malpractice was happening again.	3.17	1.35
D	24. My mind went blank.	1.79	1.26
D	25. I had amnesia for large periods of the flood.	1.82	1.24
I	26. The flood caused problems in my relationships with other people.	1.68	1.21
M	27. I had difficulty concentrating.	1.94	1.25
D	28. I felt estranged or detached from other people.	1.79	1.28
I	29. I had a vivid sense that the medical malpractice was happening all over again.	2.33	1.36
A	30. I tried to stay away from places that reminded me of the medical malpractice.	2.04	1.43

Detailed results of MMES for medical malpractice are shown in Table 5. Of all the 14 mental symptoms, less than half of the staff felt “hopeless” (39.8%), “apathy” (41.8%), “a sense of being shunned by colleagues” (21.4%), and “a sense of having been assaulted” (41.8%). On the contrary, more than 70% of the staff felt “frustration” (79.6%) and “tension” (72.4%). Of all the 15 physical symptoms, more than half felt “fatigue” (76.5%), “tiredness” (71.4%), “anxiety” (66.3%), and “tense muscles” (55.1%). Very few staff agreed with “alcohol consumption or drug use” (12.2%), “worsening of the original disease” (16.3%), “chest pains” (22.4%), and “elevated blood pressure” (22.4%). Of all the five interpersonal relationships, 64.3% indicated “offer much more medical care to avoid medical malpractice” and only 23.5% experienced “isolation from family and co-workers.”

**Table 5:** Results of medical malpractice stress syndrome (MMSS).

Variables	Yes		No	
	*N*	%	*N*	%
Mental symptoms				
Anger	49	50.0	49	50.0
Outrage	59	60.2	39	39.8
Frustration	78	79.6	20	20.4
Tension	71	72.4	27	27.6
Isolation/distrust	63	64.3	35	35.7
Negative self-image	55	56.1	43	43.9
Depression	65	66.3	33	33.7
Self-doubt	57	58.2	41	41.8
Hopelessness	39	39.8	59	60.2
Apathy	41	41.8	57	58.2
Excessive worry	50	51.0	48	49.0
Decreased interest in recreation and/or work	55	56.1	43	43.9
Sense of being shunned by colleagues	21	21.4	77	78.6
Sense of having been assaulted	41	41.8	57	58.2
Physical symptoms				
Fatigue	75	76.5	23	23.5
Inability to concentrate	45	45.9	53	54.1
Anxiety	65	66.3	33	33.7
Tiredness	70	71.4	28	28.6
Tense muscles	54	55.1	44	44.9
Insomnia	47	48.0	51	52.0
Loss of sex-drive	27	27.6	71	72.4
Alcohol consumption or drug use	12	12.2	86	87.8
Gastrointestinal upset	42	42.9	56	57.1
Chest pains	22	22.4	76	77.6
Changes in appetite	36	36.7	62	63.3
Elevated blood pressure	22	22.4	76	77.6
Headache	37	37.8	61	62.2
Decreased immunity	29	29.6	69	70.4
Worsening of the original disease	16	16.3	82	83.7
Interpersonal relationship				
Feelings of betrayal	44	44.9	54	55.1
Isolation from family and co-workers	23	23.5	75	76.5
Doubting my ability	46	46.9	52	53.1
Cannot trust patients and even everyone	44	44.9	54	55.1
Offer much more medical care to avoid medical malpractice	63	64.3	35	35.7

### Association analyses for the outcome of medical malpractice

The results of the bivariate analysis to study associations between variables and IES-R are shown in Table 6. A high total IES-R was associated with a statistical significance of young age (< 40 y/o vs. ≥ 40 y/o) and a more severe patient injury from medical malpractice (mortality > no or major injury), mainly with the constructs of intrusion and avoidance. In addition, the hyperarousal construct was associated with a statistical significance of more severe patient injury from medical malpractice (mortality > no or major injury). Patient death was significantly associated with the total IES-R and with all three constructs (intrusion, avoidance, and hyperarousal).

**Table 6:** Logistic regression for the association between possible variables and the Impact of Event Scale-Revised (IES-R).

	Total IES-R		Intrusion		Avoidance		Hyperarousal	
	Mean	SD	Mean	SD	Mean	SD	Mean	SD
Gender^t^	**1.436**		**1.305**		**1.520**		**0.883**	
Female	30.14	16.06	11.29	6.11	11.33	5.53	7.52	5.20
Male	24.00	21.66	9.17	8.16	8.48	8.10	6.35	6.09
Age (y/o)^A^	**3.666** ^*^		**3.774** ^*^		**3.496** ^*^		**2.779**	
<29	28.26	18.45	10.95	6.92	10.03	6.46	7.28	5.74
30–39	29.58	18.51	10.96	7.23	10.71	6.55	7.92	5.48
≥40	19.11	13.99	7.23	5.16	6.94	5.25	4.94	4.61
Degree^t^	**−0.331**		**−0.609**		**0.463**		**−0.863**	
Bachelor	24.23	16.34	8.86	6.16	9.64	5.68	5.73	5.30
Master, or Doctor of Philosophy	25.63	21.31	9.84	8.10	8.93	7.98	6.86	5.73
Marriage^t^	**0.518**		**0.269**		**1.004**		**0.221**	
Unmarried, divorced, widower or widow, or	26.33	15.32	9.82	5.93	9.78	5.14	6.73	5.06
Married	24.45	19.84	9.45	7.40	8.51	7.32	6.49	5.82
Executive class^t^	**−0.699**		**−0.678**		**−0.057**		**−1.378**	
No	22.15	18.08	8.46	6.82	9.00	6.35	4.69	5.58
Yes	25.80	12.83	9.80	5.08	9.11	5.61	6.89	3.50
Job^t^	**1.031**		**1.045**		**1.348**		**0.834**	
Doctor	29.75	22.79	11.00	8.51	11.25	8.57	7.50	6.42
Non-doctor	24.18	15.81	9.27	6.05	8.54	5.41	6.37	5.12
Job tenure (years) ^A^	**0.432**		**0.428**		**0.541**		**0.346**	
<2	27.83	20.73	10.38	8.10	10.25	6.94	7.21	6.16
2–10	26.15	16.85	9.94	5.88	9.29	6.05	6.91	5.62
11–20	24.12	14.97	9.71	6.69	8.12	5.54	6.29	4.18
> 20	22.35	17.00	8.30	6.09	8.30	6.38	5.74	5.20
Religion^t^	**−0.686**		**−0.411**		**−0.274**		**−1.412**	
No	24.10	20.34	9.35	7.50	8.92	7.16	5.84	6.39
Yes	26.53	14.14	9.90	5.64	9.27	5.22	7.37	4.09
Any injury of this medical malpractice to patients ^A^	**7.151** ^**^		**5.315** ^**^		**10.013** ^***^		**4.811** ^**^	
No injury	22.75	15.35	8.70	5.99	8.05	5.29	6.00	4.77
Major injury	22.73	17.50	9.27	6.78	7.91	6.75	5.55	5.68
Mortality	40.71	20.93	14.71	7.70	15.43	7.04	10.57	6.80
*Post hoc* (Scheffe)	3>1,2		3>1		3>1,2		3>1	
Did the help provided by the hospital work for you^A^	**2.019**		**1.740**		**1.720**		**2.175**	
Not at all	33.00	14.60	12.43	5.43	11.64	5.50	8.93	4.80
A little	25.02	15.89	9.44	6.12	8.98	5.52	6.59	5.04
Very much	21.10	22.33	8.29	8.31	7.71	8.24	5.10	6.40

*t*, *t*-test; A, analysis of variance, ANOVA.

The results of the bivariate analysis to study associations between variables and the SASRQ are shown in Table 7. Total SASRQ scores and nearly all constructs were not associated with variables. Dissociation was associated with the female gender and a non-doctor status. Those staff who received very much help from the hospital were associated with a significantly lower total SASRQ score, dissociation, re-experiencing of the trauma, and marked symptoms of anxiety or increased arousal.

**Table 7:** Logistic regression for the association between possible variables and Stanford Acute Stress Reaction Questionnaire (SASRQ).

	Total SASRQ		Dissociation		Re-experiencing the trauma		Avoidance of reminders of the trauma		Marked symptoms of anxiety or increased arousal		Impairment in social or occupational functioning	
	Mean	SD	Mean	SD	Mean	SD	Mean	SD	Mean	SD	Mean	SD
Gender^t^	**−1.340**		−2.702**		**−0.293**		**−1.293**		**−0.132**		**−0.962**	
Male	53.76	30.31	13.14	9.66	13.19	6.36	10.48	7.32	13.67	6.56	3.29	2.26
Female	63.43	25.16	19.35	7.95	13.64	5.49	12.74	6.27	13.88	6.92	3.82	2.22
Age (y/o)^A^	**0.478**		**1.260**		**0.086**		**0.127**		**0.459**		**0.730**	
<29	64.90	27.55	19.90	9.00	13.85	5.79	12.59	6.46	14.54	6.22	4.03	2.24
30–39	59.79	31.85	16.46	10.66	13.46	7.16	12.42	7.30	13.83	6.88	3.63	2.37
≥40	58.49	30.19	17.00	9.51	13.26	5.98	11.77	7.89	13.06	6.93	3.40	2.19
Degrees^t^	**−0.315**		**−0.688**		**0.396**		**0.114**		**−0.416**		**−0.697**	
Bachelor	59.91	31.34	16.77	10.17	14.00	6.60	12.41	7.48	13.32	6.92	3.41	2.32
Master or Doctor of Philosophy	61.78	22.13	18.38	7.54	13.41	4.40	12.21	5.93	13.99	5.47	3.79	2.02
Marriage^t^	**−0.536**		**−1.137**		**−0.077**		**−0.495**		**0.194**		**−0.963**	
Unmarried, divorced, widower or widow, or	59.62	29.81	16.82	9.69	13.49	6.02	11.87	7.29	13.98	6.51	3.47	2.40
Married	62.83	29.22	19.04	9.53	13.58	6.38	12.58	7.02	13.72	6.78	3.91	2.06
Executive class^t^	**−0.057**		**−0.100**		**0.269**		**−0.346**		**0.005**		**0.243**	
No	60.92	30.13	17.77	9.78	13.85	6.44	11.62	7.27	13.85	6.70	3.85	2.29
Yes	61.42	25.43	18.06	8.94	13.49	4.00	12.35	6.42	13.84	6.15	3.68	2.08
Job^t^	**−1.828**		−3.228**		**−0.724**		**−1.165**		**−1.130**		**−1.467**	
Doctor	50.75	26.95	12.10	7.91	12.65	6.11	10.60	7.07	12.35	6.95	3.05	2.21
Non-doctor	64.08	29.58	19.54	9.48	13.77	6.19	12.68	7.13	14.22	6.50	3.87	2.24
Job tenure (years)^A^	**0.147**		**0.344**		**0.053**		**0.135**		**0.318**		**0.780**	
<2	58.42	33.14	18.21	10.69	12.04	7.03	11.79	7.70	12.67	6.59	3.71	2.35
2–10	61.85	27.24	17.79	9.63	14.03	5.31	12.53	6.10	13.79	6.65	3.71	2.26
11–20	64.59	26.97	18.12	8.22	14.82	6.32	12.24	7.47	15.53	6.46	3.88	2.03
>21	61.30	31.79	18.09	10.05	13.43	6.33	12.35	8.11	13.87	6.82	3.57	2.41
Religion^t^	**−0.331**		**−0.271**		**−0.049**		**−0.211**		**−0.518**		**−0.852**	
No	60.37	30.69	17.76	10.15	13.51	6.25	12.10	7.19	13.49	6.82	3.51	2.36
Yes	62.35	28.40	18.29	9.18	13.57	6.12	12.41	7.15	14.18	6.43	3.90	2.14
Any injury of this medical malpractice to patients^A^	**0.938**		**1.768**		**0.200**		**1.264**		**0.866**		**1.285**	
No injury	63.58	30.56	19.08	9.91	13.75	6.40	12.56	7.30	14.30	6.85	3.88	2.27
Major injury	51.55	22.70	14.82	7.53	13.27	5.18	9.09	5.11	11.64	5.85	2.73	2.05
Mortality	57.50	27.69	19.08	8.92	13.75	5.87	12.56	7.39	14.30	5.79	3.88	2.21
Did the help provided by the hospital work for you^A^	4.211*		4.481*		5.358**		**2.535**		3.698*		**2.020**	
Not at all	66.79	29.28	18.00	9.57	16.14	5.99	13.71	8.14	15.21	6.45	3.71	2.43
A little	65.48	27.58	19.78	9.14	14.13	5.73	12.94	6.75	14.65	6.05	3.98	2.23
Very much	45.38	30.72	12.76	9.62	10.05	6.34	9.24	7.09	10.48	7.47	2.86	2.06
*Post hoc*	2>3		1,2>3						2>3			

SD, Standard deviation; t, *t*-test; A, analysis of variance, ANOVA; *p < 0.05; **p < 0.01.

## Discussion

We analyzed the impact of medical malpractice on staff, as well as the effects of care received from our institute and other associated factors on staff’s stress due to medical malpractice. When staff members encounter medical malpractice, they often experience mental and physical symptoms while still needing to maintain their professional competence to avoid further incidents. Without timely intervention, this can become a vicious cycle. Additionally, different background conditions of the staff and different types of medical malpractice can lead to different outcomes. The findings of this study highlight the importance of regular monitoring and providing appropriate care for staff based on their unique backgrounds and circumstances to ensure their well-being and protection.

In this survey, we found that scores from IES-R were not high. The highest score was the construct of intrusion (30.1). Most score levels were lower than moderate severity, typically <1.5 of the mean score, except “Item 1: Any reminder brought back feelings about it (1.58 of score)” and “Item 21: I felt watchful or on-guard (1.73 of score).” As for the results of the impact of SASRQ, the most severe construct of SASRQ was “Marked symptoms of anxiety or increased arousal.” Most were not severe SASRQ scores. For MMSE, more than 70% of staff felt “frustration” (79.6%) and “tension” (72.4%). Of all the 15 physical symptoms, more than half of the staff felt “fatigue” (76.5%), “tiredness” (71.4%), “anxiety” (66.3%), and “tense muscles” (55.1%). Very few staff agreed on “alcohol consumption or drug use” (12.2%), “worsening of the original disease” (16.3%), “chest pains” (22.4%), and “elevated blood pressure” (22.4%). Most were under mental stress, but they were able to self-adjust without substance abuse. These findings were reasonable considering that medical malpractice and likely lengthy judicial processes were stressful for the staff. Most medical staff (85.9%) spend an average of 4.7 years to prove their innocence (5). This condition is especially serious in Taiwan because of the potential for criminal prosecution and conviction. In our institute, the hospital’s care for staff was good and most of them (85.7%) felt helped by the hospital. Even under mental stress, our staff were still able to handle the situation.

Medical malpractice can be committed by various healthcare providers, including doctors, nurses, nursing assistants, administrators, and others who provide care for patients on a daily basis. Authorities of the medical institute should be aware of the basic aspects of medical professional liability and should help to control damages from medical malpractice (30). In this study, we found that female staff (all nurses) and non-doctors showed higher scores of SASRQ. Younger staff encountering more severe patient injuries also showed similar high scores. In summary, young female staff at non-doctor and non-administrative positions encountering severe patient injury were those showing the highest risks of stress and anxiety. The results are reasonable since medical staff with longer job tenures (also with a better chance to be administrators) were more familiar with and more educated about medical malpractice. As for doctors and non-doctors, physicians were more used to facing medical malpractice and the impact on them was less. Notably, doctors at this institute receive regular training on how to handle potential disputes, which may have influenced their responses. Medical disputes for non-doctor medical staff have been better studied in recent years (31–34). Nurses are undoubtedly the healthcare professionals who have the most contact with patients and, as a result, are most likely to become involved in incidents. With respect to age, it is noteworthy that older medical staff members are more likely to have experienced medical malpractice incidents in the past and, thus, may have developed a greater ability to manage the associated stress and emotional impact. Consequently, our findings suggest that older staff members were less likely to have higher scores on the SASRQ. In conclusion, hospital policymakers may wish to direct their attention toward younger non-doctor staff members and provide them with early interventions in order to prevent serious incidents of medical malpractice from occurring. This proactive approach would be of great benefit to minimize negative impacts on medical staff members. In our institute, the chief of the department and social worker initially reach out to the affected staff members to offer support, which includes a review of the incident, counseling, and mental health support. If any legal concerns were raised, our hospital’s legal team is also available to provide further support to the staff members. Based on the results of this study, it is evident that this support system has proven to be effective.

This study is subject to certain limitations. Firstly, we did not collect data on the specialties of the physicians involved in the incidents. Secondly, we lacked information on the socio-demographic characteristics of the plaintiffs. Thirdly, the frequency with which medical staff members had previously experienced incidents of medical malpractice was not recorded. Fourthly, the sample size was small and not well-balanced in terms of participant characteristics. Finally, it is possible that some staff members may have forgotten certain details of the incident due to the time lapse between the occurrence of the incident and their completion of the questionnaire. However, we believe that any such bias is likely to be minor since all medical staff members claimed that they would not forget the emotional impact of such incidents.

## Conclusion

Our study underscores the importance of hospital authorities regularly monitoring staff members’ responses to incidents of medical malpractice. The provision of mechanisms for reporting near misses, coupled with ongoing staff training, can help to mitigate the incidence of adverse events associated with healthcare practices. With effective and timely interventions, it is possible to break the vicious cycle of medical malpractice. Based on the findings of this study, hospital authorities should develop a strategic plan aimed at preventing incidents of medical malpractice by prioritizing the care of young, non-doctor, and non-administrative medical staff members.

## Data availability statement

The original contributions presented in the study are included in the article/supplementary material, further inquiries can be directed to the corresponding authors.

## Ethics statement

The studies involving human participants were reviewed and approved by CE18097A. The patients/participants provided their written informed consent to participate in this study. Written informed consent was obtained from the individual(s) for the publication of any potentially identifiable images or data included in this article.

## Author contributions

S-FT, T-FY, and C-HC: conceptualization and data curation. C-LW and T-FY: methodology. Y-YH and T-FY: software. C-LW and C-HC: validation and writing—review and editing. S-FT and T-FY: formal analysis and writing—original draft preparation. Y-YH, P-YL, A-CY, Y-HYah, and C-MH: investigation. C-LW and Y-HYou: resources. C-LW, T-FY, and C-HC: supervision and funding acquisition. S-FT and Y-HYah: project administration. All authors contributed to the article and approved the submitted version.

## Funding

This study was supported by grant TCVGH-CTUST1077702 from the Taichung Veterans General Hospital and Central Taiwan University of Science and Technology.

## Conflict of interest

The authors declare that the research was conducted in the absence of any commercial or financial relationships that could be construed as a potential conflict of interest.

## Publisher’s note

All claims expressed in this article are solely those of the authors and do not necessarily represent those of their affiliated organizations, or those of the publisher, the editors and the reviewers. Any product that may be evaluated in this article, or claim that may be made by its manufacturer, is not guaranteed or endorsed by the publisher.
